# Association of QTc Interval and V4-S Wave With Appropriate ICD Therapy in Hypertrophic Cardiomyopathy

**DOI:** 10.3389/fcvm.2022.882662

**Published:** 2022-05-12

**Authors:** Nixiao Zhang, Sijing Cheng, Hongxia Niu, Min Gu, Hui Peng, Zhijun Sun, Xi Liu, Yu Deng, Xuhua Chen, Wei Hua

**Affiliations:** ^1^Department of Cardiology, Cardiovascular Center, Beijing Friendship Hospital, Capital Medical University, Beijing, China; ^2^Cardiac Arrhythmia Center, Fuwai Hospital, National Center for Cardiovascular Diseases, Chinese Academy of Medical Sciences and Peking Union Medical College, Beijing, China

**Keywords:** hypertrophic cardiomyopathy, implantable cardioverter-defibrillator, appropriate therapy, electrocardiogram, sudden cardiac death (SCD)

## Abstract

**Background:**

Ventricular arrhythmias in patients with hypertrophic cardiomyopathy (HCM) may lead to sudden cardiac death (SCD). We aimed to investigate the relationship between electrocardiogram (ECG) indicators and the risk of appropriate implantable cardioverter-defibrillator (ICD) therapy in HCM.

**Methods:**

The HCM patients receiving ICD implantation were enrolled consecutively. QT interval correction (QTc) was calculated using Bazett's formula. Long or deep S wave in V4 lead was defined as duration time >50 ms and/or voltage amplitude >0.6 mV. The endpoint in our study was at least one ICD appropriate therapy triggered by ventricular tachyarrhythmia (VT) or ventricular fibrillation (VF), including anti-tachyarrhythmia pacing (ATP) and electrical shock.

**Results:**

A total of 149 patients with HCM (mean age 53 ± 14 years, male 69.8%) were studied. Appropriate ICD therapies occurred in 47 patients (31.5%) during a median follow-up of 2.9 years. Cox regression analysis showed that long or deep S wave in V4 lead [hazard ratio (HR) 1.955, 95% confidence interval (CI) 1.017–3.759, *P* = 0.045] and QTc interval (HR 1.014, 95% CI 1.008–1.021, *P* < 0.001) were independent risk factors for appropriate ICD therapy. The ROC showed that the optimal cut-off point value for the QTc interval to predict the appropriate ICD therapy was 464 ms, and the AUC was 0.658 (95% CI 0.544–0.762, *P* = 0.002). The AUC for S wave anomalies in V4 lead was 0.608 (95% CI 0.511–0.706, *P* = 0.034). We developed a new model that combined the QTc interval and S wave anomalies in V4 lead based on four patient groups. Patients with QTc ≥464 ms and long or deep V4-S wave had the highest risk of developing appropriate ICD therapy (log-rank *P* < 0.0001). After adding QTc interval and V4-S wave anomalies into the HCM-risk-SCD model, the prediction effect of the new model was significantly improved, and the NRI was 0.302.

**Conclusions:**

In this HCM cohort, QTc and S wave anomalies in V4 lead were found to be significant and strong predictors of the risk of appropriate ICD therapy. Patients with QTc ≥464 ms and long or deep S wave had the highest risk. After QTc interval and V4-S wave anomalies adding to the HCM-risk-SCD model, the prediction effect is significantly improved.

## Introduction

The pathophysiological characteristics of hypertrophic cardiomyopathy (HCM) are cardiomyocyte hypertrophy, interstitial fibrosis and disorder of the cardiomyocyte fiber arrangement, which increase the risk of sudden cardiac death (SCD) (1). These structural changes may explain the abnormality in the electrical activity of the left ventricle, including depolarization and repolarization on the surface electrocardiogram (ECG) (1, 2). The ECG patterns showed non-specific change in 75 to 95% of HCM patients (3). Recent studies found that the severity of ECG abnormalities was associated with structural and functional findings in cardiac magnetic resonance (CMR), including left ventricular mass, myocardial hypertrophy, and fibrosis (4–7). However, whether these ECG abnormalities are related to electrical instability and could identify patients with a higher risk of ventricular arrhythmia (VT) or SCD is still not determined. The HCM risk-SCD model recommended by 2014 European Society of Cardiology guidelines was widely used in clinical practice. The HCM risk-SCD model included family history of SCD, maximal left ventricular wall thickness, syncope, non-sustained ventricular tachyarrhythmias (NSVT), age, left atrial diameter (LAD), and maximal left ventricular outflow tract gradient (LVOTG) (8). In 2019, the enhanced American College of Cardiology/American Heart Association guideline incorporated novel high-risk markers, such as extensive late gadolinium enhancement (LGE), systolic dysfunction and left apical ventricular aneurysms (9). These models provide essential value for the selection of patients suitable for implantable cardioverter defibrillator (ICD).

However, there are some controversies regarding the effect of these two assessment methods (10, 11). These two methods were not ideal for predicting the risk of SCD when applied to Chinese patients (11). Therefore, it is complicated to determine the risk stratification of the patients and make a decision on ICD implantation.

Standard 12-lead ECG is a simple, reproducible and inexpensive test that could be used by cardiologists and general practitioners and is one of the non-invasive tools for HCM patients (12, 13). Fragmented of the QRS complex (fQRS) on the ECG was the manifestation of abnormal cardiac depolarization and was associated with myocardial fibrosis (14). Studies have found that fQRS was the independent risk factor of arrhythmic events in ischemic cardiomyopathy and non-ischemic cardiomyopathy (15). It was also the marker of the substrate for spontaneous ventricular fibrillation in Brugada syndrome patients and was the diagnostic marker of arrhythmogenic right ventricular dysplasia (16, 17). QT prolongation was the arrhythmogenic substrate in long QT syndrome and drug-induced QT prolongation (18). Several studies evaluating the role of QTc prolongation in HCM did not have homogenous findings (4, 19, 20). HCM patients could have huge T-wave inversion in ECG (TWI). However, it is still unknown whether the TWI is secondary to depolarization abnormality or repolarization abnormality. The prediction value of TWI for SCD risk was still controversial (21, 22). Recently, Lyon et al. (23) used mathematical modeling and computational clustering to analyze the 12-lead Holter ECGs for the prediction of the risk of SCD. This study included 85 patients and 38 healthy volunteers and had found that primary TWI may increase SCD risk in HCM. Our aim was to investigate whether 12-lead ECG could predict ICD therapy in a Chinese HCM cohort.

## Methods

### Study Population

This is a retrospective single-center observational study. One hundred and sixty four HCM patients who successfully implanted ICD in Fuwai hospital between June 2007 and August 2020 were included. Patients had the indication for ICD when they had at least one of the following risk factors (24): (1) history of resuscitation of cardiac arrest, (2) history of premature HCM- related sudden death in one or more first-degree relatives, (3) documented non-sustained ventricular fibrillation, (4) documented ventricular events or unexplained syncope, or decision regarding the risk status and ICD implantation was made at the discretion of the managing cardiovascular specialists (usually involving electrophysiologists) using established risk stratification for primary or secondary prevention of SCD. Fifteen patients were excluded as follows: (1) need for ventricular pacing (*n* = 5), (2) lost to follow-up (*n* = 3), (3) low-quality ECG (*n* = 7). The study conformed to the principles of the Declaration of Helsinki. Written informed consent was obtained from each patient.

### Diagnosis of HCM

HCM was defined as left ventricular thickness ≥15 mm (or ≥13 mm if family history of HCM was present) in one or more ventricular myocardial segments measured by echocardiography, cardiac magnetic resonance (CMR) or computed tomography (CT) in the absence of another cause of hypertrophy (25).

### ECG Diagnosis and Analysis

We retrospectively analyzed the 12-lead ECG of HCM patients prior to ICD implantation. ECG was measured at 25 mm/s paper speed, 10 mm/mV amplitude and 0.05-100 Hz filter setting. We only analyzed clearly recorded ECG. The ECG signals were calculated as the mean of three consecutive beats by an ECG caliper through an electronic medical recording system. The signals were measured and averaged by two researchers (NX Zhang and SJ Cheng) in the same ECG, who were blinded to the patients' medical status.

The signals we analyzed included heart rate, complete left bundle branch block (cLBBB), complete right bundle branch block (cRBBB), atrioventricular block (AVB), left ventricular high voltage (LVHV), fQRS, deep S wave or long S wave in V4, TWI, P wave duration, PR duration, QRS duration and corrected QT interval (QTc). The signals except the S wave were measured in lead II, V5 or V6.

**Figure 1:**
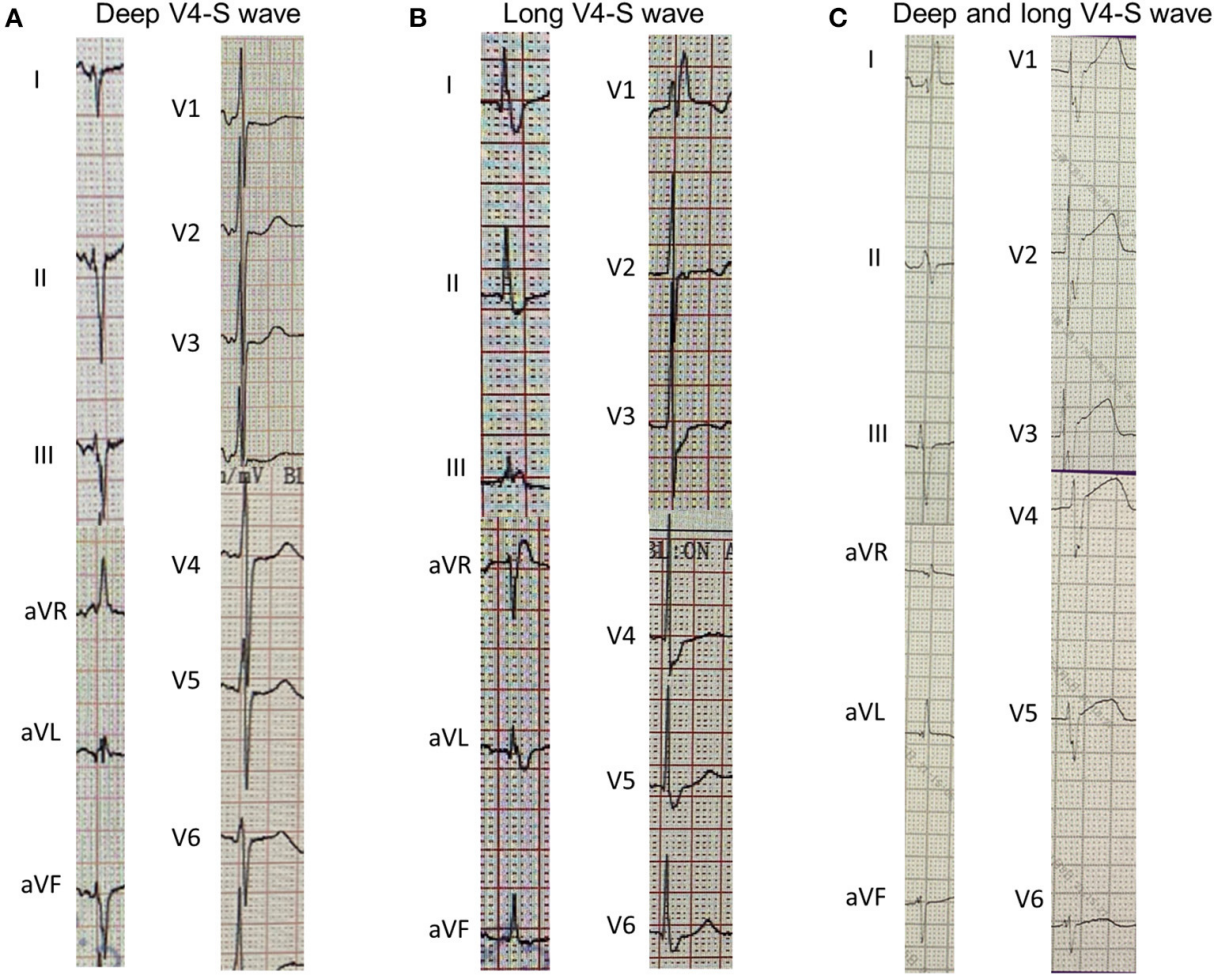
Three cases of long or deep V4-S wave in 12-lead electrocardiogram. **(A)**, V4-S wave depth >0.6 mV; **(B)**, V4-S wave duration >50 ms; **(C)**, V4-S wave is wide and deep.

The cLBBB was defined as QRS duration >120 ms, QS or rS in lead V1, wide R wave with the absence of Q wave in lead I or lead V6, notched or slurred QRS in more than two leads of V1, V2, V5, V6, I, aVL. The cRBBB was defined as a late R (R') in V1 or V2, with slurred wide S wave in leads I and V5/V6 and QRS duration >120 ms. LVHV was based on Cornell criteria (S wave amplitude in V3 plus R wave amplitude in aVL ≥20 mm in female and ≥28 mm in male) or Sokolow-Lyon criteria (S wave amplitude in V1 or V2 plus R wave amplitude in V5 or V6 ≥35 mm). fQRS was defined as RSR' morphology (an additional R wave or notching in the nadir of the S wave) in more than two contiguous leads. Long or deep S wave was defined as prolongation of S wave (S wave duration >50 ms in V4 lead) and/or S wave amplitude deepening (>0.6 mV) (Three cases of patients with long or deep S wave were listed in Figure 1). TWI was defined as the amplitude of T wave >0.1 mV, and huge TWI was defined as amplitude >1.0 mV. PR duration was measured between the start of the P wave and the beginning of the QRS complex. QRS duration was measured between the start of the QRS complex and the J point. QT interval was measured between the start of the QRS complex and the end of the T wave. QTc was calculated according to Bazett's formula (QTc = QT/(RR)^1/2^). The end of the T wave was defined as the intersection between the tangent of the descending limb of the T wave and the isoelectric line. If there was a U wave after the T wave, the end of the T wave was the intersection of the tangent of the steepest part of the T wave descending limb and the isoelectric line.

### ICD Therapy and Follow-Up

We regularly implanted transvenous ICD. In patients without venous access, with the difficult crossing of the tricuspid valve, or after tricuspid valve replacement, S-ICD was implanted. Type and programming parameters of ICD were based on the decision-making of patients and doctors. The appropriate ICD therapy was defined as delivering anti-tachycardia pacing (ATP) or shock for sustained ventricular tachycardia (VT) or ventricular fibrillation (VF). We documented the occurrence of ICD therapy for VT or VF via stored electrograms and reviewed by an experienced electrophysiologist. For patients without program information, we obtained the ICD therapy information via the medical calls. The censored date was January 31th, 2021.

### HCM-risk-SCD Model

HCM-risk-SCD model was calculated as follows: 5-year risk = 1-0.998 exp (prognosis index), where prognosis index = [0.15939858^*^Maximal wall thickness (mm)]– [0.00294271^*^Maximal wall thickness^2^(mm^2^)] + [0.826391195^*^NSVT] + [0.0259082^*^LAD (mm)]+ [0.00446131^*^maximal LVOTG (mmHg)] + [0.71650361^*^ unexplained syncope] + [0.4583082^*^ family history of SCD] – [0.01799934^*^age at evaluation (years)] (8).

### Statistical Analysis

All analysis were done using SPSS version 25.0 and R version 3.6. Continuous variables were presented with mean and standard deviation or median and interquartile range according to distribution. Student's *t*-test or Mann-Whitney *U*-test was used to compare groups as appropriate. Categorical variables were presented with count and percentage, and Chi-square was used to compare. Receiver operating characteristic (ROC) curves were generated to evaluate the prediction efficacy of factors for SCD and calculate the best cutoff value of QTc. The prediction efficacy was presented with the area under the curve (AUC), and the larger the AUC was, the greater the prediction efficacy was. We used the Cox regression model to select the prediction factor for SCD. We included all variables that showed a trend toward an association with effect at uni-variable Cox regression analysis (*P* < 0.1). And we also included the use of amiodarone as it could affect the QTc. Pearson correlation test was used to analyze the relationship between QTc and MWT, and the Spearman correlation test for the relationship between QTc and LVOTG. Net reclassification index (NRI) was used to evaluate the change of prediction efficacy. *P*-value <0.05 were considered significant.

## Results

### Characteristics of the Patient

One hundred forty-nine HCM patients were enrolled in our study. Among these, 9 of 149 patients (6.0%) had apical hypertrophic cardiomyopathy (APHCM), 102 patients (68.5%) implanted ICD for primary prevention, while 47 patients (31.5%) implanted ICD for secondary prevention. Among these patients, 23 (15.4%) had coronary heart disease. The mean HCM- SCD risk score was 5±3%. For ECG indicators, 37 patients (24.8%) had LVHV; 19 patients (12.8%) had fQRS; 73 patients (49%) had long or deep S wave; and 65 patients (43.6%) had TWI, of whom six patients (4.0%) had huge TWI. The mean duration of P wave, PR duration, QRS complex and QTc interval was 105 ± 36 ms, 168 ± 51 ms, 111 ± 27 ms, and 445 ± 45 ms, respectively (Table 1).

**Table 1:** Baseline characteristics of HCM patients with and without appropriate ICD therapy.

**Variable**	**All patients** **(*n* = 149)**	**Patients without appropriate ICD therapy (*n* = 102)**	**Patients with appropriate ICD therapy (*n* = 47)**	***P***
**Demographic characteristics**				
Age (y)	53 ± 14	54 ± 14	51 ± 19	0.288
Male (%)	104 (69.8)	72 (70.6)	32 (68.1)	0.848
BMI (kg/m^2^)	25 ± 3	25 ± 3	25 ± 4	0.277
SBP (mmHg)	119 ± 14	119 ±± 15	120 ± 14	0.818
DBP (mmHg)	72 ± 9	72 ± 9	72 ± 9	0.984
**Comorbidity**				
Diabetes mellitus (%)	25 (16.8)	19 (18.6)	6 (12.8)	0.482
Hypertension (%)	55 (36.9)	38 (37.3)	17 (36.2)	>0.999
AF (%)	43 (28.9)	35 (34.3)	8 (17.0)	0.030
Coronary artery disease (%)	23 (15.4)	18 (17.6)	5 (10.6)	0.335
**ICD prevention**				0.451
Primary prevention (%)	102 (68.5)	72 (70.6)	30 (63.8)	-
Secondary prevention (%)	47 (31.5)	30 (29.4)	17 (36.2)	-
Family history of SCD	38 (25.5)	25 (24.5)	13 (27.7)	0.690
Syncope, *n* (%)	88 (59.1)	60 (58.8)	28 (59.6)	>0.999
NSVT, *n* (%)	60 (40.3)	41 (40.2)	19 (40.4)	>0.999
APHCM, *n* (%)	9 (6.0)	7 (6.9)	2 (4.3)	0.72
HOCM, *n* (%)	28 (18.8)	22 (21.6)	6 (12.8)	0.261
ASA, *n* (%)	2 (1.3)	1 (1.0)	1 (2.1)	0.533
MORROW, *n* (%)	3 (2.0)	3 (2.9)	0	0.552
SCD risk score, %	5 ± 3	5 ± 3	5 ± 3	0.562
**Echocardiography**				
LAD, mm	42 ± 6	43 ± 6	41 ± 6	0.143
LVMT, mm	22 ± 6	22 ± 6	21 ± 5	0.268
Maximal LVOTG, mmHg	6.8 (4.8-10.6)	6.8 (4.8-16.0)	6.8 (4.8-10.2)	0.562
LVEDD, mm	47 ± 8	47 ± 7	49 ± 8	0.110
LVEF, %	60 ± 11	60 ± 12	59 ± 10	0.474
RVD, mm	21 ± 3	21 ± 3	21 ± 3	0.869
Ventricular aneurysm, (%)	4 (2.7)	3 (2.9)	1 (2.1)	>0.999
**ECG indicators**				
Heart rate, bpm	66 ± 14	67 ± 14	66 ± 13	0.937
cLBBB, *n* (%)	4 (2.7)	4 (3.9)	0	0.308
cRBBB, *n* (%)	8 (5.4)	2 (2.0)	6 (12.8)	0.020
LVHV, *n* (%)	37 (24.8)	28 (27.5)	9 (19.1)	0.314
fQRS, *n* (%)	19 (12.8)	12 (11.8)	7 (14.9)	0.604
S wave abnormality, *n* (%)	73 (49.0)	43 (42.2)	30 (63.8)	0.021
**TWI**, ***n*** **(%)**				0.327
TWI>0.1, <1.0 mV	65 (43.6)	48 (47.1)	17 (36.2)	-
Giant TWI	6 (4.0)	5 (4.9)	1 (2.1)	-
P wave duration, ms	105 ± 36	106 ± 39	102 ± 29	0.592
PR interval, ms	168 ± 51	163 ± 53	179 ± 45	0.099
QRS complex, ms	111 ± 27	107 ± 22	118 ± 33	0.058
QTc, ms	445 ± 45	436 ± 36	464 ± 56	0.003
**Drug usage**, ***n*** **(%)**				
β-block	139 (93.3)	95 (93.1)	44 (93.6)	>0.999
Amiodarone	78 (52.3)	56 (54.9)	22 (46.8)	0.382
ACEI/ARB	60 (40.3)	43 (42.2)	17 (36.2)	0.590

*Values are presented as mean ± SD or median (IQR), or as n (%).*

*HCM, hypertrophic cardiomyopathy; ICD, implantable cardioverter-defibrillator; BMI, body mass index; SBP, systolic blood pressure; DBP, dilated blood pressure; AF, atrial fibrillation; SCD, sudden cardiac death; NSVT, non-sustained ventricular tachyarrhythmia; APHCM, apical hypertrophic cardiomyopathy; HOCM, hypertrophic obstructive cardiomyopathy; ASA, alcohol septal ablation; LAD, left atrial diameter; LVMT, left ventricular maximum thickness; LVOTG, left ventricular outflow tract gradient; LVEDD, left ventricular end-diastolic diameter; LVEF, left ventricular ejection fraction; RVD, right ventricular diameter; ECG, electrocardiogram; cLBBB, complete left bundle branch block; cRBBB, complete right bundle branch block; LVHV, left ventricular high voltage; TWI, T wave inversion; ACEI/ARB, angiotensin converting enzyme inhibitor/ angiotensin receptor blocker*.

### Follow-Up

The median follow-up was 2.9 years (IQR: 1.7–5.6 years). Forty Seven patients (31.5%) received ICD appropriate therapy. The characteristics of patients who received appropriate ICD and who did not receive appropriate ICD therapy are presented in Table 1. The baseline characteristics and echocardiography parameters were similar between the two groups, except for a higher proportion of patients with atrial fibrillation (AF) without appropriate ICD therapy (34.3 vs. 17.0%, *P* = 0.03). There was a higher proportion of patients with long or deep S wave in V4 among patients with appropriate ICD therapy (63.8 vs. 42.2%, *P* = 0.021). Moreover, patients with appropriate ICD therapy had longer QTc (464 ms ± 56 ms vs. 436 ± 36 ms, *P* = 0.003).

### Cox Regression Analysis

In univariate Cox regression analysis, long or deep S wave was significantly associated with appropriate ICD therapy (HR 2.197, 95%CI 1.197–4.035, *P* = 0.011). QTc at baseline and RBBB were also significantly associated with ICD appropriate therapy (HR 1.014, 95%CI 1.008–1.021, *P* < 0.001; HR 3.196, 95%CI 1.342–7.613, *P* = 0.009, respectively). In multivariate analysis, we included AF, RBBB, long or deep S wave, QTc, HCM-risk SCD score and history of amiodarone. We did not include age, family history of SCD, or LVOTG due to the presence of these parameters in the HCM-risk-SCD score. After adjusting for confounding factors, long or deep S wave and QTc were independent predictors for ICD appropriate therapy (HR 1.955, 95%CI 1.017–3.759, *P* = 0.045; HR 1.014, 95%CI 1.008–1.021, *P* < 0.001, respectively). The HCM-risk-SCD score was also an independent risk factor in our cohort (HR 1.110, 95%CI 1.003–1.229, *P* = 0.043) (Table 2).

**Table 2:** Univariable and multivariable models for predictors of appropriate ICD therapies.

**Variable**	**Univariable analysis**		**Multivariable analysis**	
	**Hazard ratio (95%CI)**	***P***	**Hazard ratio (95%CI)**	***P***
AF	0.472 (0.220–1.011)	0.053	0.459 (0.203–1.040)	0.062
HCM-risk-SCD	1.024 (0.932–1.126)	0.620	1.110 (1.003–1.229)	0.043
cRBBB	3.196 (1.342–7.613)	0.009	2.022 (0.813–5.026)	0.130
S wave abnormality	2.197 (1.197–4.035)	0.011	1.955 (1.017–3.759)	0.045
QTc	1.013 (1.007–1.019)	<0.001	1.014 (1.008–1.021)	<0.001
Amiodarone	0.770 (0.432–1.374)	0.377	0.893 (0.471–1.693)	0.729

*ICD, implantable cardioverter-defibrillator; AF, atrial fibrillation; HCM, hypertrophic cardiomyopathy; SCD, sudden cardiac death; cRBBB, complete right bundle branch block*.

### Relationship Between QTc and MWT and LVOTG

QTc interval was not correlated with MWT (r=0.095, P=0.251) nor LVOTG (*r* = – 0.070, *P* = 0.394).

### The Prediction Efficacy of the Combination of QTc and V4-S Wave

**Figure 2:**
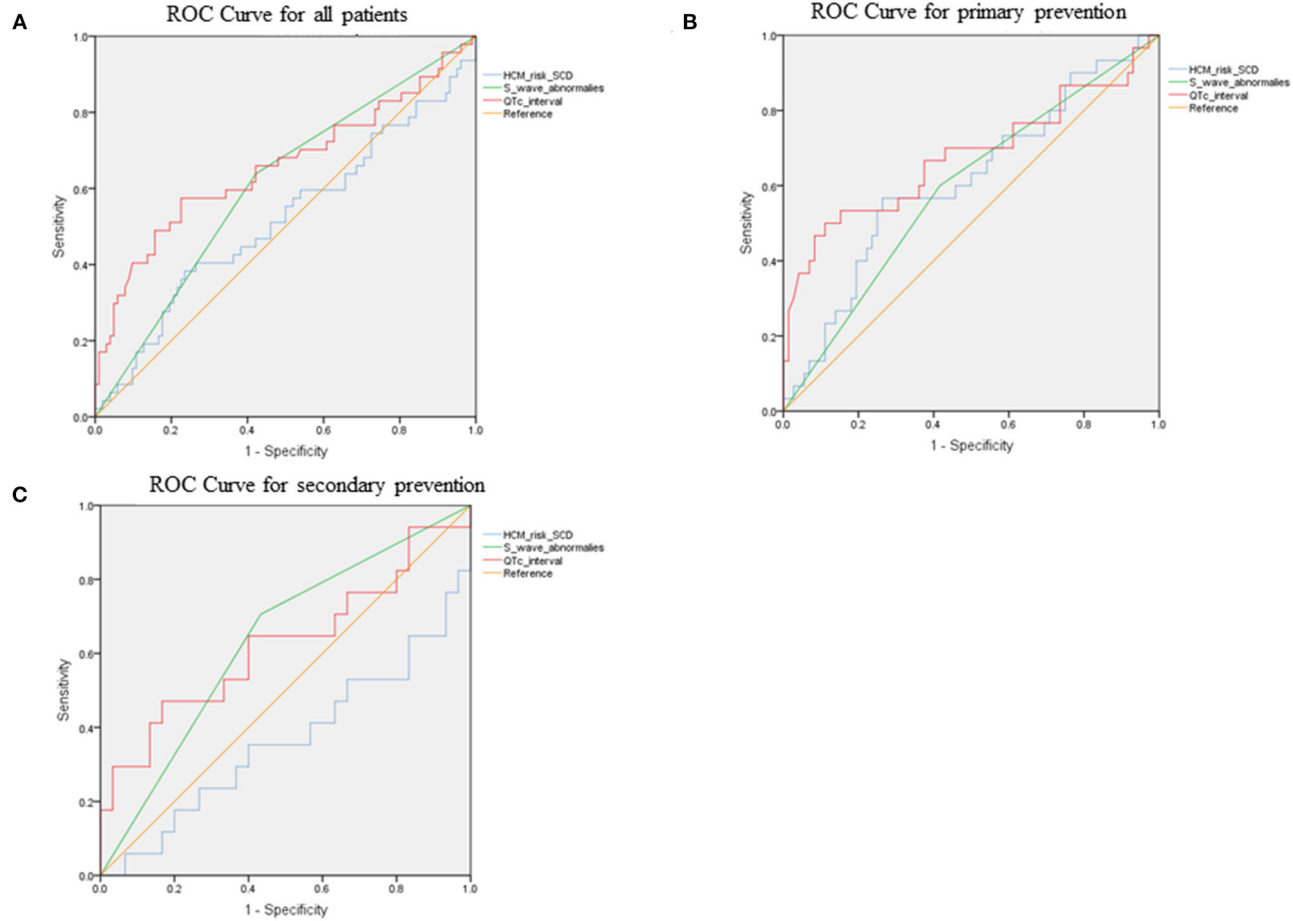
The ROC curves for predicting appropriate ICD therapies by HCM-risk-SCD model, QTc interval, and V4-S wave. **(A)** for all patients, **(B)** for primary prevention, and **(C)** for secondary prevention. ROC, receiver operating curve; ICD, implantable cardioverter-defibrillator; HCM, hypertrophic cardiomyopathy; SCD, sudden cardiac death.

**Figure 3:**
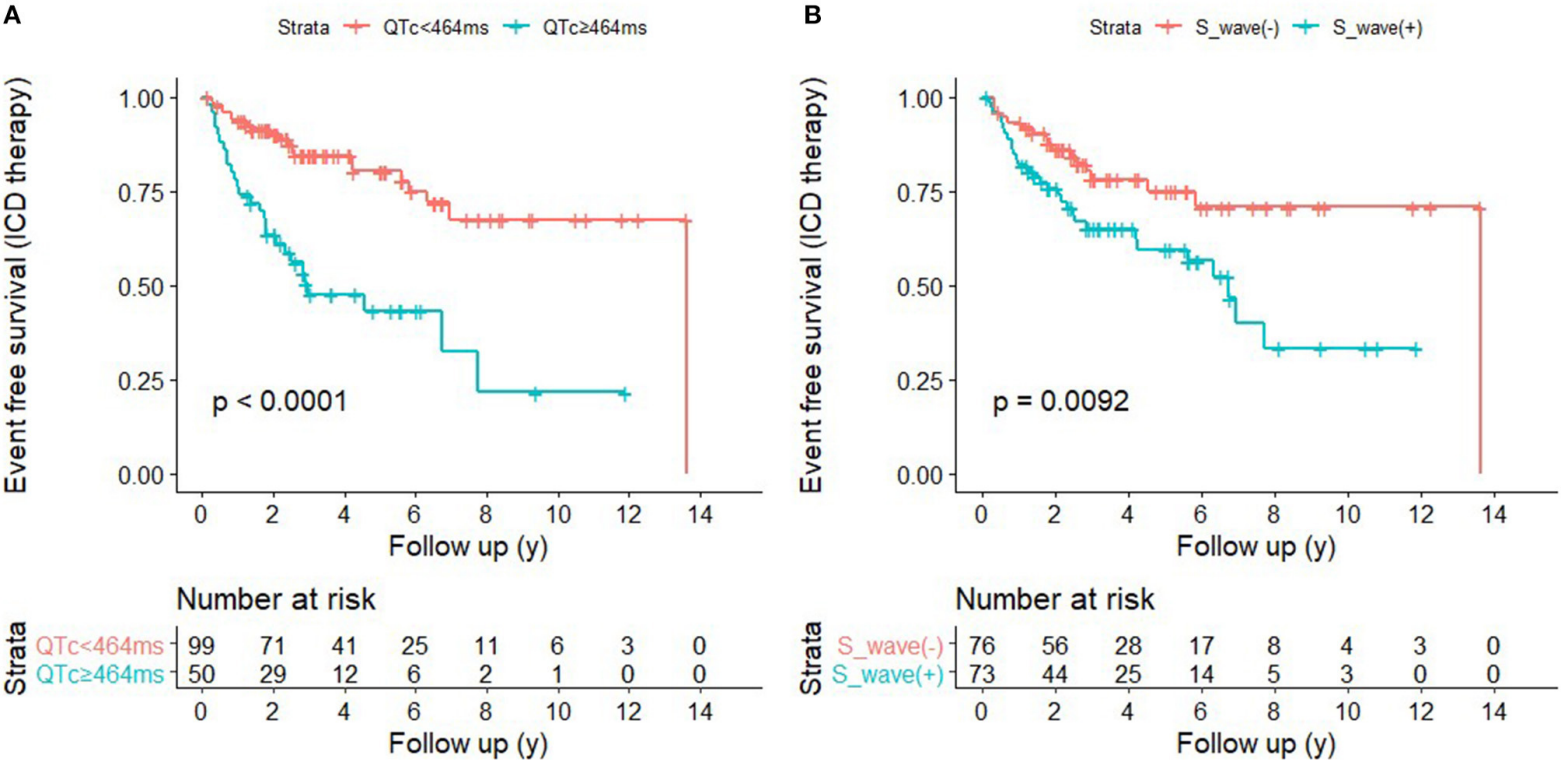
Kaplan-Meier survival analysis based on QTc interval **(A)** and wide or deep V4- S wave **(B)**.

ROC curves were presented in Figure 2 to investigate the prediction efficacy of QTc and long or deep S wave in V4. In ROC curves, AUC in HCM-risk-SCD score for ICD appropriate therapy was 0.517 (95%CI 0.412–0.623, *P* = 0.735). As shown in Figure 2A, the two ECG indicators achieved a high prediction value, with an AUC of 0.608 (95%CI 0.511–0.706, *P* = 0.034) for S wave anomalies and 0.658 (95%CI 0.554–0.762, *P* = 0.002) for QTc with the optimal cutoff value of 464 ms. We demonstrated that the risk of appropriate ICD therapy was higher in patients with QTc≥464 ms than QTc <464 ms (Log-rank *P* < 0.0001) (Figure 3A). Similarly, the risk of ICD appropriate therapy was higher in patients with long or deep S wave in V4 (Log-rank *P* = 0.009) (Figure 3B).

### Association of S Wave and QTc With Appropriate ICD Therapy

In this study, 73 patients (49.0%) presented long or deep S wave. Compared with patients without long or deep S wave, patients with long or deep S wave had a higher rate of fQRS (20.5 vs. 5.3%, *P* = 0.006), longer QRS duration (118 ms ± 29 ms vs. 103 ms ± 22 ms, *P* = 0.002), and lower usage of amiodarone (43.8 vs. 60.5%, *P* = 0.041). There was no significant difference in QTc duration, HCM-risk-SCD score and rate of ICD implantation for secondary prevention between the two groups. The rate of patients with long or deep S wave receiving appropriate ICD therapy was higher than patients without long or deep S wave (41.1 vs. 22.4%, *P* = 0.014).

There were 99 patients (66.4%) with QTc <464 ms, while 50 patients (33.6%) had a QTc ≥464 ms. The rate of ICD implantation for secondary prevention was significantly higher in patients with QTc≥464 ms (23/50, 46%) than in patients with QTc <464 ms (24/99, 24.2%). There were significant differences in QRS complex duration (119 ms ≥ 30 ms vs. 107 ≥ 24 ms, *P* = 0.017) and usage of amiodarone (34/50 (68%) vs. 44/99 (44.4%), *P* = 0.007) in patients with QTc ≥464 ms compared with patients with QTc <464 ms. The rate of long or deep S wave was similar in the two groups (24/50 (48%) in QTc ≥464 ms vs. 49/99 (49.5%) in QTc <464 ms, *P* = 0.863). The rate of patients with QTc ≥464 ms receiving appropriate ICD therapy was higher compared with patients with QTc <464 ms (27/50 (54%) vs. 20/99 (20.2%), *P* < 0.0001) (Table 3).

**Table 3:** Patients stratified according to QTc interval and S wave anomalies.

**Variable**	**S wave abnormality (-)** **(*n* = 76)**	**S wave abnormality (+)** **(*n* =73)**	**QTc <464 ms** **(*n* = 99)**	**QTc ≥464 ms (*n* = 50)**	*P*-value^*^	***P*-value^#^**
**Demographic characteristics**						
Age (y)	54 ± 14	52 ± 16	54 ± 15	52 ± 17	0.401	0.573
Male (%)	49 (64.5)	55 (75.3)	73 (73.7)	31 (62.0)	0.149	0.141
BMI (kg/m^2^)	25 ± 3	25 ± 4	25 ± 3	24 ± 4	0.576	0.089
SBP, mmHg	119 ± 15	119 ± 13	120 ± 14	118 ± 15	0.835	0.630
DBP, mmHg	72 ± 9	72 ± 9	71 ± 9	73 ± 9	0.656	0.221
**Comorbidity**						
Diabetes Mellitus (%)	16 (21.1)	9 (12.3)	17 (17.2)	8 (16.0)	0.154	0.857
Hypertension (%)	25 (32.9)	30 (41.1)	39 (39.4)	16 (32.0)	0.300	0.377
AF (%)	25 (32.9)	18 (24.7)	27 (27.3)	16 (32.0)	0.267	0.548
Coronary heart disease (%)	8 (10.5)	15 (20.5)	12 (12.1)	11 (22.0)	0.091	0.115
Family history of SCD, *n* (%)	21 (27.6)	17 (23.3)	27 (27.3)	11 (22.0)	0.543	0.486
Syncope, *n* (%)	48 (63.2)	40 (54.8)	58 (58.6)	30 (60.0)	0.299	0.868
NSVT, n (%)	34 (44.7)	26 (35.6)	43 (43.4)	17 (34.0)	0.256	0.268
APHCM, *n* (%)	3 (3.9)	6 (8.2)	6 (6.1)	3 (6.0)	0.321	>0.999
HOCM, *n* (%)	15 (19.7)	13 (17.8)	20 (20.2)	8 (16.0)	0.763	0.535
ASA, *n* (%)	1 (1.3)	1 (1.4)	0	2 (4.0)	>0.999	0.111
MORROW, *n* (%)	2 (2.6)	1 (1.4)	1 (1.0)	2 (4.0)	>0.999	0.261
HCM-risk-SCD, %	5 ± 3	5 ± 3	5 ± 3	5 ± 3	0.185	0.768
**ICD prevention**, ***n*** **(%)**					0.487	0.007
Primary prevention	54 (71.1)	48 (65.8)	75 (75.8)	27 (54.0)	-	-
Secondary prevention	22 (28.9)	25 (34.2)	24 (24.2)	23 (46.0)	-	-
**Echocardiography**						
LAD, mm	42 ± 6	42 ± 7	42 ± 7	42 ± 5	0.963	0.964
LVMT, mm	22 ± 5	22 ± 6	22 ± 5	23 ± 6	0.453	0.260
Maximal LVOTG, mmHg	6.8 (4.8-9.0)	6.8 (4.9-11.3)	6.8 (4.8-14.4)	6.8 (4.8-9.0)	0.638	0.595
LVEDD, mm	47 ± 8	48 ± 7	48 ± 7	47 ± 8	0.204	0.599
LVEF, %	61 ± 11	59 ± 12	60 ± 11	58 ± 11	0.364	0.232
RVD, mm	21 ± 3	22 ± 3	22 ± 3	21 ± 3	0.298	0.177
Ventricular aneurysm	4 (5.3)	0	2 (2.0)	2 (4.0)	0.120	0.602
**ECG indicators**						
Heart rate, bpm	66 ± 13	66 ± 14	65 ± 14	69 ± 12	0.983	0.067
LBBB, *n* (%)	2 (2.6)	2 (2.7)	3 (3.0)	1 (2.0)	>0.999	>0.999
RBBB, *n* (%)	1 (1.3)	7 (9.6)	5 (5.1)	3 (6.0)	0.031	>0.999
LVHV, *n* (%)	17 (22.4)	20 (27.4)	25 (25.3)	12 (24.0)	0.478	0.867
fQRS, *n* (%)	4 (5.3)	15 (20.5)	14 (14.1)	5 (10.0)	0.006	0.474
S wave abnormality, *n* (%)	-	-	49 (49.5)	24 (48.0)	-	0.863
**TWI**, ***n*** **(%)**					0.323	0.864
TWI >0.1, <1.0 mV	33 (43.4)	32 (43.8)	45 (45.5)	20 (40.0)	-	-
Giant TWI	5 (6.6)	1 (1.4)	4 (4.0)	2 (4.0)	-	-
P wave duration, ms	110 ± 43	99 ± 25	101 ± 36	111 ± 34	0.096	0.199
PR interval, ms	161 ± 54	175 ± 46	166 ± 48	172 ± 56	0.147	0.571
QRS duration, ms	103 ± 22	118 ± 29	107 ± 24	119 ± 30	0.002	0.017
QTc, ms	443 ± 40	447 ± 50	421 ± 28	493 ± 32	0.584	<0.0001
QTc <464 ms	50 (65.8)	49 (67.1)	-	-	0.863	-
**Drug usage**, ***n*** **(%)**						
β-block	70 (92.1)	69 (94.5)	93 (93.9)	46 (92.0)	0.794	0.655
Amiodarone	46 (60.5)	32 (43.8)	44 (44.4)	34 (68.0)	0.041	0.007
ACEI/ARB	27 (35.5)	33 (45.2)	43 (43.4)	17 (34.0)	0.228	0.268
Appropriate ICD therapy, *n* (%)	17 (22.4)	30 (41.1)	20 (20.2)	27 (54.0)	0.014	<0.0001

* *P represented the comparison between patients with S wave abnormality and without S wave abnormality.*

# *P represented the comparison between Patients with QTc ≥464 ms and QTc <464 ms.*

*Values are presented as mean ± SD or median (IQR), or as n (%).*

*HCM, hypertrophic cardiomyopathy; ICD, implantable cardioverter-defibrillator; BMI, body mass index; SBP, systolic blood pressure; DBP, dilated blood pressure; AF, atrial fibrillation; SCD, sudden cardiac death; NSVT, non-sustained ventricular tachyarrhythmia; APHCM, apical hypertrophic cardiomyopathy; HOCM, hypertrophic obstructive cardiomyopathy; ASA, alcohol septal ablation; LAD, left atrial diameter; LVMT, left ventricular maximum thickness; LVOTG, left ventricular outflow tract gradient; LVEDD, left ventricular end-diastolic diameter; LVEF, left ventricular ejection fraction; RVD, right ventricular diameter; ECG, electrocardiogram; cLBBB, complete left bundle branch block; cRBBB, complete right bundle branch block; LVHV, left ventricular high voltage; TWI, T wave inversion; ACEI/ARB, angiotensin converting enzyme inhibitor/angiotensin receptor blocker*.

**Figure 4:**
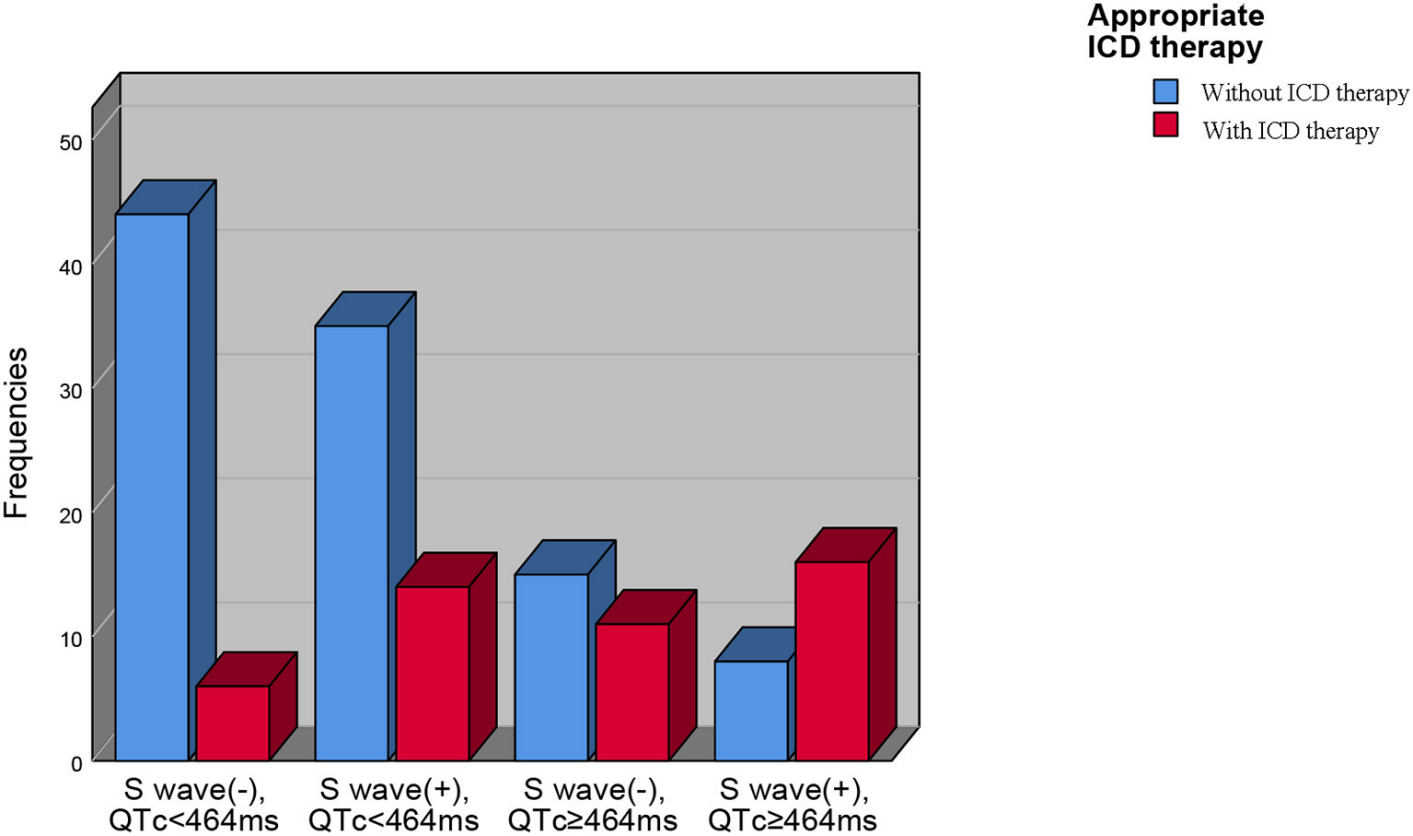
The prevalence of appropriate ICD therapies in four subgroups based on QTc interval and V4-S wave. ICD, implantable cardioverter-defibrillator.

**Figure 5:**
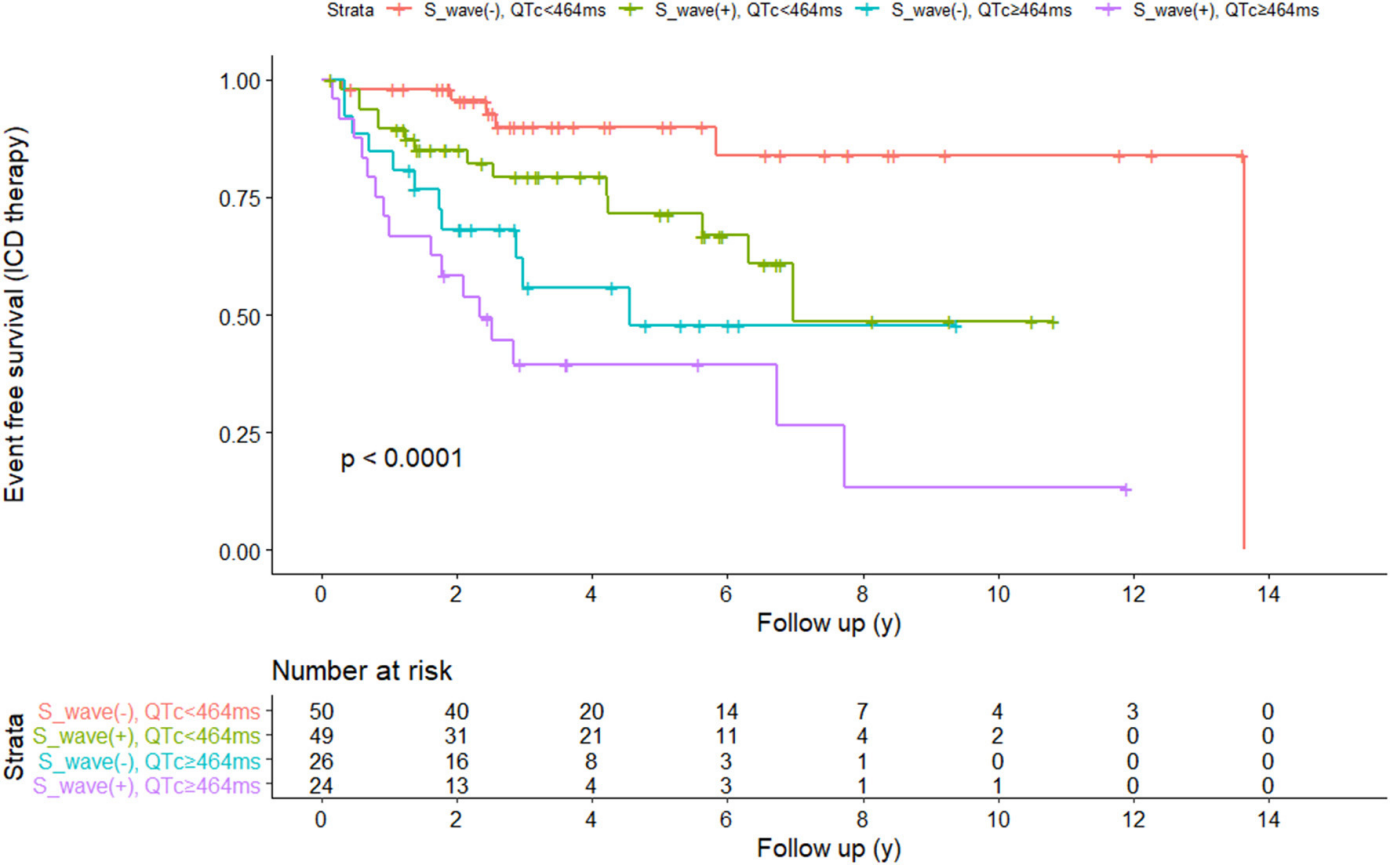
Kaplan-Meier survival analysis curves were compared among the four subgroups.

We divided our patients into four groups according to QTc and long or deep S wave:(1) without long or deep S wave (-) and QTc <464 ms (*n* = 50); (2) with long or deep S wave (+) and QTc <464 ms (*n* = 49); (3) without long or deep S wave (-) and QTc ≥464 ms (*n* = 26); (4) with long or deep S wave (+) and QTc ≥464 ms (*n* = 24). There were 6 (12.0%), 14 (28.6%), 11 (42.3%), and 16 (66.7%) patients receiving appropriate ICD therapy, respectively (Figure 4). Kaplan-Meier analysis demonstrated that there was a significant difference among the four groups (*P* < 0.0001) (Figure 5). Pairwise comparison analysis showed that there were differences between group1 and group 2 (*P* = 0.026), group 1 and group 3 (*P* = 0.002), group 1 and group 4 (*P* < 0.0001), and group 2 and group 4 (*P* = 0.004). The highest appropriate ICD therapy rate was in group 4.

### Improvement of Prediction Efficacy of HCM-risk-SCD After Inclusion of New Parameters

**Figure 6:**
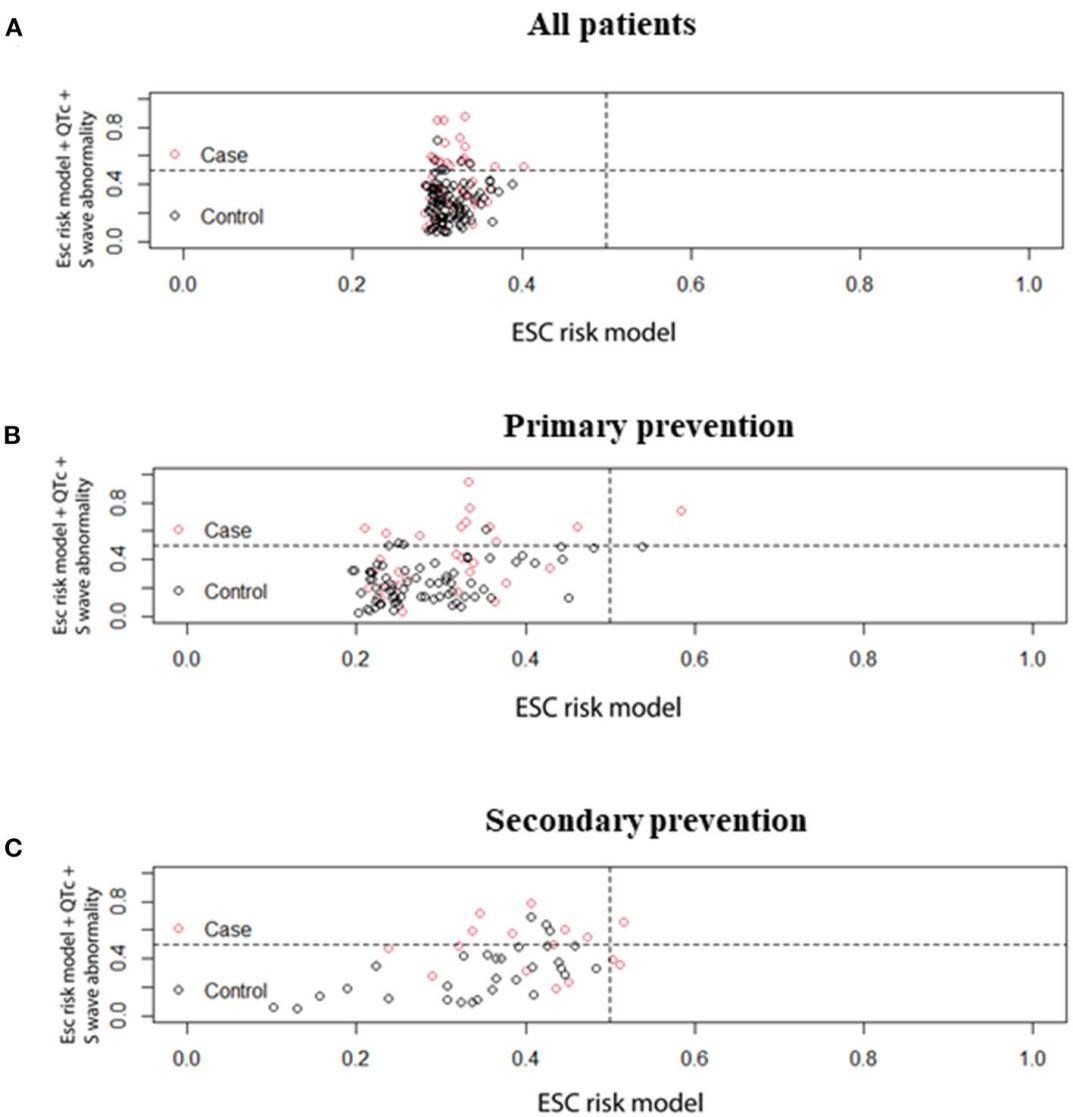
Comparison of prediction effect of HCM-risk-SCD model before and after adding QTc and V4-S wave simultaneously. **(A)** for all patients, **(B)** for primary patients, and **(C)** for secondary patients. HCM, hypertrophic cardiomyopathy; SCD, sudden cardiac death.

We added long or deep S wave in V4 to the HCM-risk-SCD model, and the C- statistics of the new model 1 was 0.625 (95%CI 0.526–0.723, *P* = 0.015). The new model improved reclassification with the net reclassification index (NRI) of 0.011. Then, we added QTc into the HCM-risk-SCD model. The C-statistics of the new model 2 was 0.659 (95%CI 0.552-0.765), and the NRI was 0.193. Finally, we added long or deep S wave in V4 and QTc in the HCM-risk-SCD model simultaneously. The C-statistics of new model 3 was 0.702 (95%CI 0.607–0.796), and the NRI was 0.302 (Figure 6A).

### Subgroups With Primary and Secondary Prevention

Figures 2B,C demonstrated ROC curves of S-wave anomalies and QTc interval stratified according to primary and secondary prevention.

In the primary prevention patients, S wave anomalies and QTc yielded AUC of 0.592 (95%CI 0.470–0.713, *P* = 0.146) and 0.678 (95%CI 0.548–0.808, *P* = 0.06), respectively. While the HCM-risk-SCD model yield AUC of 0.613 (95%CI 0.490–0.735, *P* = 0.074) as shown in Figure 6B, the addition of long or deep S wave and QTc into the HCM-risk SCD model showed reclassification improvement (NRI 0.306).

In the secondary prevention patients, S wave anomalies and QTc yielded AUC of 0.636 (95%CI 0.471–0.801, *P* =0.124) and 0.624 (95%CI 0.444–0.803, *P* = 0.163), respectively. While the HCM-risk-SCD model yield AUC of 0.363 (95%CI 0.188–0.537, *P* = 0.121, Figure 6C). And the addition of long or deep S wave and QTc into the HCM-risk SCD model resulted in reclassification improvement (NRI 0.135, Figure 6C).

## Discussion

In this study, we demonstrated that: (1) long or deep S wave in V4 and QTc ≥464 ms were independent factors of appropriate ICD therapy in HCM patients implanted with ICD; (2) Patients with QTc ≥464 ms and long or deep V4-S wave had the highest risk of appropriate ICD therapy; (3) addition of QTc and long or deep S wave improved the prediction efficacy of HCM-risk-SCD model (NRI: 0.302).

### QTc in HCM Patients

Approximately 75–95% of HCM patients had abnormal 12-lead ECG (3). However, not all abnormal ECG patterns were associated with ventricular arrhythmias. QT interval is defined from the beginning of the QRS complex to the end of the T wave and contains the sum of the duration of myocardium depolarization and repolarization. Several studies have demonstrated that QTc was associated with severity of left ventricular hypertrophy, LVOTG, potential pathogenic mutations (mutations may affect sodium and potassium channels involved in depolarization and repolarization), and activity of sympathetic nerve (19, 20, 26). Furthermore, myocardial fibrosis, myocardium arrangement disorder and/or microvascular ischemia may affect the QTc in HCM patients. Many studies investigated the influence of QTc on the prognosis of HCM patients (20, 27). In our study population, 33.6% of patients had a QTc ≥464 ms, compared to the 13% of patients in a previous study (27). The numerically higher prevalence in our study may be due to the inclusion the patients with the usage of amiodarone which could prolong the QTc. A previous study has demonstrated that the patients with QTc ≥460 ms had a 3-fold increased risk of VA or SCD (27). Gray et al. (28) found that the risk for appropriate ICD therapy was >3-fold in patients with QTc ≥464 ms compared with patients without QTc prolongation after adjustment for LVWT and sex. Similarly, QTc prolongation had an arrhythmogenic effect in our study. Maron et al. demonstrated that QTc prolongation and greater QT dispersion were present in HCM patients but were not predictors for SCD (29). This result may be due to differences in population, genetic background, and outcomes between the two studies.

A possible explanation for the lack of correlation between QTc interval and the increase in left ventricular wall thickness is that prolonged QTc interval is associated with myocardial fibrosis. Studies have found that, in addition to myocardial hypertrophy, interstitial fibrosis and myocardial fibrosis may lead to prolonged QTc interval, which is the substrate of arrhythmia (30). Riza Demir et al. (4) enrolled 74 HCM patients and underwent CMR. They found that QTc in HCM patients with myocardial fibrosis was longer than in patients without fibrosis (455 ± 38 ms vs. 430 ± 29ms, *P* = 0.002). And QTc could predict LGE in CMR (OR 1.024, 95%CI 1.007–1.040, *P* = 0.004). On the contrary, Delcrè et al. (19) demonstrated no correlation between QTc prolongation and prevalence of LGE (*p* = 0.08). We did not assess the relationship between myocardial fibrosis, QTc and appropriate ICD therapy. Thus, this potential mechanism needs further investigation.

The prolonged QTc interval of HCM patients may also be related to genetic factors. HCM and long QT syndrome (LQTS) are heart diseases caused by abnormal proteins encoded by two sets of disease-causing genes (31, 32). Studies have shown that these two sets of inheritance may be related to each other. HCM patients may have some mutations in LQTS, which may lead to the prolongation of the QT interval and the occurrence of arrhythmia events (31). Nevertheless, we did not perform genetic testing on patients, so any genetic evaluation of the LQTS gene was not possible.

### V4-S Wave in HCM Patients

S wave is the terminal part of the QRS complex and represents the depolarization vector of the right ventricle and the posterosuperior late left ventricular. Arrhythmogenic cardiomyopathy (ACM) mainly involves the right ventricle (delay in the right ventricular activation or conduction), in which ECG may manifest as a delay in terminal activation delay (TAD) in the right chest lead. TAD >55 ms is a predictor of right ventricular dilatation and dysfunction (33). Right ventricular insufficiency was a prediction factor of SCD in patients with ACM (34). Recently, more and more studies have begun to explore the effect of right ventricular involvement on HCM patients. Wu et al. compared the right ventricular function and the exercise tolerance of 76 HCM patients and 30 healthy people and found that a higher proportion of right ventricular dysfunction and reduction of contractile reserve in HCM patients (35). Seo et al. included 256 HCM patients and found that right ventricular involvement (CMR showed that the free wall thickness of the right ventricle ≥7 mm) was related to abnormal left ventricular structure and biventricular dysfunction. Right ventricular involvement and impaired right ventricular strain were predictors of composite endpoints (all-cause death, cardiac transplantation, and cardiovascular hospitalization) (36). Lyon et al. used machine learning algorithms to regroup HCM patients based on baseline characteristics to investigate the risk of arrhythmia and the severity of myocardial hypertrophy. Researchers found that the algorithm automatically divided HCM into three groups based on QRS morphology: group 1 with normal QRS morphology; group 2 with low R wave amplitude in lead V4 and large S wave amplitude; and group 3 with low R wave amplitude in lead V4-V6, wide S wave, and left axis deviation. However, the study found no significant differences in the risk of arrhythmia and the severity of myocardial hypertrophy (23). And the study did not further explore the relationship between the differences in ECG of the three groups and SCD.

In this study, we could not explore the effect of these factors on the appropriate treatment of ICD due to the absence of CMR examination. Although there was no significant difference in the anteroposterior diameter of the right ventricle between patients with appropriate ICD therapy and patients without appropriate ICD therapy, more subtle changes in the structure and function of the right ventricle, such as myocardial fibrosis, right ventricular strain, etc. could be found. Some subclinical impairments of the right ventricular myocardium may appear before the reduced right ventricular function (37). However, subclinical impairments in the right ventricle were likely to affect the ECG vector. Therefore, we measured the S wave in lead V4 and found that the long or deep V4-S wave was an independent predictor of the appropriate ICD therapy. And after it was added to the HCM-risk-SCD model, the prediction effect of the model was significantly increased. In univariate Cox regression, both long or deep S wave and RBBB were correlated with ICD therapy, but in multivariate Cox regression, the correlation between long or deep V4-S wave and appropriate ICD therapy was independent of RBBB. Most patients with RBBB were accompanied with deep and wide S-wave in lead V4, so to a certain extent long or deep S-wave could represent or contain most RBBB. Therefore, RBBB was not significant in multivariate regression.

### Subgroup Analysis

In this study, we did not restrict the analysis to patients in primary prevention but also included patients with secondary prevention. Prior study has shown that not all patients implanted ICD for secondary prevention would receive ICD therapy during follow-up (38), thus it was also essential to identify patients in this indication who had a higher risk of appropriate therapy. In our study, there was no interaction between indications for ICD and whether to receive ICD therapy. And analysis according to the indication demonstrated that S wave anomalies and QTc improved the risk stratification in both subgroups.

## Limitations

This study has several limitations. Firstly, this was a single-center retrospective study with a small sample size. A prospective trial with a large sample size was needed to verify the relationship between the long or deep S wave in V4 and the appropriate ICD therapy. Secondly, the patients in this study did not routinely undergo pathogenic genetic tests. Thirdly, in our research V4-S wave was a categorical variable defined based on manual measurement, and we did not measure the precise time duration and amplitude of the S wave. More research is needed to measure and explore the optimal cut-off value for prediction accurately. Fourthly, as CMR data are not available for this study, no further conclusion can be drawn on whether MRI, especially LGE could be related to appropriate ICD therapy in this population. Fifthly, approximate 50% of patients in our study were on therapy with amiodarone. Our results might only suggest that longer QTc could predict SCD, but cannot suggest a widely applicable cut-off value. However, it is indeed impossible to determine a fixed QTc cut-off value in HCM populations with different amiodarone utilization rates. Lastly, appropriate ICD therapy, associated with the programming of the device, is not equal to SCD. Nevertheless, the findings of this study are still meaningful.

## Conclusion

QTc duration and long or deep V4-S wave were independent predictors of appropriate ICD therapy in HCM patients with ICD. Patients with QTc ≥464 ms and long or deep V4-S wave had the highest risk of appropriate ICD therapy. The addition of QTc duration and long or deep V4-S wave to HCM-risk-SCD improved the prediction efficacy. These two ECG parameters might help better stratify HCM patients with ICD.

## Data Availability Statement

The raw data supporting the conclusions of this article will be made available by the authors, without undue reservation.

## Ethics Statement

The studies involving human participants were reviewed and approved by the Ethics Committee of Fuwai Hospital. The patients/participants provided their written informed consent to participate in this study.

## Author Contributions

NZ and SC contributed to conception and design of the study and organized the database. NZ performed the statistical analysis and wrote the first draft of the manuscript. SC wrote sections of the manuscript. All authors contributed to manuscript revision, read, and approved the submitted version.

## Conflict of Interest

The authors declare that the research was conducted in the absence of any commercial or financial relationships that could be construed as a potential conflict of interest.

## Publisher's Note

All claims expressed in this article are solely those of the authors and do not necessarily represent those of their affiliated organizations, or those of the publisher, the editors and the reviewers. Any product that may be evaluated in this article, or claim that may be made by its manufacturer, is not guaranteed or endorsed by the publisher.
